# Altitude does not protect against SARS‐CoV‐2 infections and mortality due to COVID‐19

**DOI:** 10.14814/phy2.14922

**Published:** 2021-06-10

**Authors:** Laura Cardenas, Valeria Valverde‐Bruffau, Gustavo F. Gonzales

**Affiliations:** ^1^ Laboratories of Investigation and Development (LID) Faculty of Sciences and Philosophy Universidad Peruana Cayetano Heredia Lima Peru; ^2^ High Altitude Research Institute Universidad Peruana Cayetano Heredia Lima Peru

## CONFLICT OF INTEREST

The authors declare no competing interests.

## AUTHOR CONTRIBUTIONS

L.C.—Data analysis and the writing of the manuscript text. V.V‐B.—Data analysis and the writing paper. G.F.G.—Advised the data analysis and interpretation and contributed to writing the text. All authors revised the text during the review process.

To the Editor,

We read with interest the paper from Millet et al. (2021), which discuss reports suggesting that high‐altitude residence may be beneficial in the novel coronavirus disease (COVID‐19). They conclude that no evidence‐based knowledge is presently available on whether and how altitude/hypoxia may prevent, treat, or aggravate COVID‐19. They suggest that the reported lower incidence and mortality of COVID‐19 in high‐altitude places observed by others (Stephens et al., 2021) remain to be confirmed. Stephens et al. (2021) study two groups: one living in counties located at altitudes less than 914 m and the other at altitudes higher than 2133 m.

Castagnetto et al. (2020) assessing data at district level have demonstrated that at different altitude ranges from 0 to <1000 m, 1000 to 2500 m, and 2500 to 4700 m in Peru, the negative association with altitude was observed only in the group ranging from 0 to <1000 m. If analysis is done using all altitudes from 0 to 4700 m a negative association is observed between altitude and cases with SARS‐CoV‐2. Fernandes et al. (2021) assessing 154 cities in Brazil located between 5 and 1135 m. They observe a negative association between altitude and cases of SARS‐CoV‐2. Altitudes higher 1135 m were not assessed.

Cano‐Pérez et al. (2020) in Colombia analyzed data from 70 selected municipalities with altitudes between 1 and 3180 m. They suggest that living at high altitude can reduce the impact of COVID‐19, especially the case fatality rate. However, when the total of 1122 municipalities in the same country were analyzed no altitude gradient that is protective against SARS‐CoV‐2 infection or COVID‐19 mortality could be demonstrated (Valverde‐Bruffau et al., 2020).

In this letter we relate cases, deaths, and CFR by COVID‐19 to altitude using the district approach in Peru and Colombia. At all, 2881 districts in Peru and Colombia have been analyzed, varying in altitudes from 2 to 4705 m. Data from 1,745,498 cases and 65,797 COVID‐19 deaths were included in the analysis. Data is publicly available and as it is not possible to identify individuals in this data, then anonymity is guaranteed.

We have assessed COVID‐19 cases, deaths, and case fatality rates (CFR) in three gradients of different altitudes: from 2 to <1500 m, 1500 to <2500 m, and 2500 m to 4700 m. We have also controlled in the analysis by geographical area. When the entire altitude range was assessed, it was observed that the increase in altitude correlated with a decrease in positive cases of SARS‐CoV‐2 and COVID‐19 deaths (*p* < 0.001), but positively with CFR/1000 (*p* = 0.0004).

When evaluated at the three gradients of different altitude ranges, in the multivariate analysis is appreciated that the differences between altitudes are observed in the range from 2 to <1500 m (*n* = 1273 districts). In this range (from 2 to <1500 m), the altitude increase was inversely associated with the positive cases of SARS‐CoV‐2 (*R* ^2^ = 0.03; −0.69 ± 0.09; Coefficient beta ± standard error; *p *< 0.001), deaths (*R* ^2^ = 0.09; −0.04 ± 0.003; *p* < 0.001) and the CFR (*R* ^2^ = 0.01; −0.0013 ± 0.00420; *p* = 0.001) per COVID‐19.

Between 1500 and <2500 m (*n* = 500 districts), cases were positively associated with altitude (*R* ^2^ = 0.01; 0.38 ± 0.16; coefficient beta ± standard error; *p* = 0.018), deaths (*R* ^2^ = 0.01; 0.013 ± 0.006; *p* = 0.015), and CFR (*R* ^2^ = 0.002; 0.008 ± 0.017; *p* = 0.631).

Between 2500 and 4700 m (*n* = 1108 districts), no association was observed between the increase in altitude and cases (*R* ^2^ = 0.003; −0.08 ± 0.06; Coefficient beta ± standard error; *p* = 0.17), between altitude and deaths (*R* ^2^ = 0.001; −0.005 ± 0.003; *p* = 0.09), and between altitude and CFR (*R* ^2^ = 0.003; 0.0027 ± 0.01; *p* = 0.785).

According to this study, when the entire range of altitudes is used to correlate with positive cases of SARS‐CoV‐2, it is possible to obtain an inverse relationship, but this association is limited to the range between 2 and <1500 m. We confirmed the same pattern when deaths due to COVID‐19 and CFR were evaluated.

From this, it is suggested that altitude itself does not constitute protection against infection or CFR, and that the relationship between cases, deaths, and CFR at altitudes of 2 to <1500 m is due to other causes not related to environmental factors clearly associated with increased altitude such as hypoxia, increased UV radiation, and aridity, among others (Millet et al., 2021). In this altitude range (2 to <1500 m), we have observed that at a lower geographical area there are a greater number of deaths/100,000 inhabitants and CFR and this can partially explain this association between altitude and COVID‐19 between 2 and <1500 m. At lower altitudes and particularly in capital cities, there is greater flow of people, higher vehicular traffic, greater agglomeration that facilitate contagion with SARS‐CoV‐2. The highest number of cases at lower altitudes in the range of 0–1500 m could also be due to high air pollution in large cities (Vasquez‐Apestegui et al., 2020).

When all altitudes (2–4700 m) are assessed in a single analysis, an inversely proportional correlation with altitude is observed as reported in other studies (Castagnetto et al., 2020; Segovia‐Juarez et al., 2020). This relationship is apparently an artifact as only this negative correlation is evident at altitudes between 0 and <1500 m. From 1500 to 4700 m, no association of positive cases with altitude is observed even though variables such as partial oxygen pressure, humidity, solar and cosmic radiation and humidity, and production of vitamin D change proportionally as altitude increase (Pun et al., 2020); further production of the angiotensin 2‐converting enzyme, a carrier molecule for the entry of SARS‐CoV‐2 virus into the host cell, has also been suggested (Millet et al., 2021; Srivastava et al., 2020). Our data suggest that none of these factors protect people living in high altitudes to reduce SARS‐CoV‐2 infections or the severity of COVID‐19 disease, and people must not be confident and engage in behaviors that could reduce the effectiveness of protective measures such as vaccination, physical distancing, washing hands, mask wearing, and avoiding large gatherings of people.

It is likely that confounding factors such as availability of services to treat, diagnose, or test for COVID‐19 as well as for determining the cause of death, among others—all of which are less available at higher altitudes sites perhaps gave rise to the earlier, now‐apparently erroneous report of a "protective" effect of high‐altitude residence.

It is also important to highlight that we have presented retrospective and data‐base based observations. This is just association, like all other related publications. We cannot establish causal association although other literatures have somewhat led that kind of narrative.

In conclusion, altitude, especially above 1500 m, is not a protective factor for SARS‐CoV‐2 virulence or lethality. Understanding the epidemiology of COVID‐19 is increasingly important in guiding control measures. Overall, this study should be followed by larger, better designed efforts, to increase our understanding of the impact of altitude in situations of comorbidities as obesity, diabetes mellitus, hypertension and excessive erythrocytosis.
